# Pulpless Teeth

**Published:** 1885-10

**Authors:** 

**Affiliations:** Burlington, Member of the Faculty of the Dental Department of the State University.


ARTICLE II.

### PULPLESS TEETH.

BY DR. WILSON, OF BURLINGTON, MEMBER OF THE FACULTY OF THE DENTAL DEPARTMENT OF THE STATE UNIVERSITY.

**[Extracts of a paper read before the Iowa Dental Association at its late annual meeting.]**

A pulpless tooth is not necessarily a dead tooth, but a dead tooth is, of course, a pulpless tooth. The adjectives "pulpless" and "dead" are not, therefore, synonymous, although frequently so used, especially by medical writers. Let us note the marked distinction between the two. A pulpless tooth may be a part of the living organism—a dead tooth has its nutritive supply entirely cut off, and it is in every sense a foreign body—it is dead and inert. The former may be restored to health and usefulness—the latter should always be condemned as a nuisance that cannot be abated without the use of the forceps. * * * *

Having thus briefly called attention to the fact that the dentine and cementine derive their vitality from independent sources—that the life of the one is not dependent upon the life of the other—that a pulpless tooth is not necessarily a dead tooth—we are prepared to consider, understandingly, the subject of this paper. It may, however, seem like presumption on the part of the writer, in thus offering the foregoing to an intelligent body of dentists, when every student of dentistry at the close of his junior year should fully understand the facts above stated. But I am led to a consideration of this subject from articles entitled, "Dead Teeth in the Jaws," that have appeared, from time to time, during the last two years, in the New York *Medical Record*, and as those articles come from high sources in the medical profession, they deserve more than passing notice. The able editor of that journal, and Dr. Samuel Sexton, a distinguished oculist and aurist of New York City, being the principal writers referred to.

The *Medical Record* of October 4, 1884, contains a report from the aural service of Dr. Sexton, entitled, "Pain in the Ears due to Irritation in the Jaws." He describes a number of cases of otalgia in which he found the lesion to be in diseased teeth.

He goes on to say that "since dentistry had become such a popular business, and diseased teeth had been so carefully retained in the jaws, nervous diseases about the head were becoming alarmingly common."

The same number of the above journal contained an editorial on "Dead Teeth in the Jaws," which read as follows: "Perhaps the time is near at hand when medical men should be themselves better informed concerning diseases of the jaws and mouth, rather than refer the ailments of this region to individuals whose limited knowledge of medicine does not prevent them from 'treating' dead teeth long after their presence in the jaws has given rise to alveolar abscesses and neuralgias more or less painful. It would not be strange if in the course of events, the day would soon come when all teeth without pulps, and hence in process of more or less rapid decay, as well as those which the deposit of tartar, or other cause, had become entirely divested of periosteal nourishment, would be promptly condemned as unfit to remain in the jaws, regarded in fact as foreign bodies liable to give rise, not only to cerebral irritation and disease in the organs of special sense, through the propagation of local disturbances in the mouth to the regions mentioned, but to endanger likewise the general health through purulent matter discharged into the mouth from alveolar abscesses, to be continuously swallowed for a long time, or, indeed, in some instances, to be absorbed and thus produce septicæmic poisoning. It is certainly gratifying to note the establishment of instruction in oral surgery in some of the medical schools, and it is to be hoped that this subject will receive the attention its importance demands."

Dr. Sexton cites the readers of the *Record* to eight cases of otalgia resulting from diseased teeth. I have no doubt but a majority of the dentists before me to-day have met with almost that number of cases in practice every week; nor do you find it a difficult thing to render prompt relief, and that, too, in a large majority of cases, without the use of the forceps. And I believe that I am warranted in saying that in at least three-fourths of the cases met with in our practice, we find the reflex pain in the ears due to exposed living pulps, and not to "dead teeth in the jaws."

That diseased teeth do cause reflex trouble, not only in the head, but frequently in more remote parts of the body, is a fact well-known to every competent dentist. I am glad that Dr. Sexton has at last discovered the fact, that diseased teeth do frequently cause reflex pain in the ears, and in other neighboring parts, and that alveolar abscesses very often cause catarrhal affections of the maxillary sinus and of the nasal passages, and that diseased teeth will endanger the general health. It is to be regretted, however, that the doctor has found it necessary to charge this unfortunate state of affairs to the ignorance of dental practitioners, who are in no way responsible for but few of the many cases met with in practice, for there can be no doubt but a very large majority of the teeth causing the troubles above referred to have never received any treatment whatever at the hands of dentists, and because Dr. Sexton has discovered that in certain cases pulpless teeth (or dead teeth as he calls them), has caused the ailments above referred to by Dr. Sexton, there can be no doubt. Every dentist of any considerable experience can enumerate such experiences by the score, and the medical profession has only been too slow to recognize the facts discovered by Dr. Sexton.

The only difficulty with these medical gentlemen is, that they have drawn very erroneous conclusions from the important discoveries they have made. Their limited knowledge of the minute structure of the dental tissue, and the source from which each derives its life, is manifested by the erroneous statements upon which they have based their arguments, and then after arguing from false premises, Dr. Sexton says: "In regard to the treatment of pulpless teeth, the practice in vogue seems the reverse of precedures founded on well-established surgical principles." And in an editorial of the same issue we are informed that the treatment of diseased teeth is carried, to what "the medical minds regard as a dangerous extreme."

That some members of our profession have been over zealous in their efforts to save all diseased pulps alive, there can be no doubt. We will occasionally meet with an enthusiast in our profession who will say, "I have no use for forceps, I never extract teeth." I have heard that statement made on the floor of the Iowa State Dental Association.

That incurable diseased teeth should not be tolerated in the jaws does not admit of discussion. Good common sense ought to settle that question. And again, there are extremists who never devitalize diseased pulps, no matter how badly exposed, but "doctor them up," and stupify them, and then bury them in a living grave. Much evil has grown out of this practice.

Some one has said that to cap a badly exposed pulp is to create a slumbering volcano, and he might well have added that such volcanoes have but a limited time to slumber. Gentlemen, there are in our own country ten thousand volcanoes belching forth—not pure molten lava—but impure gases and putrescent matter of the most sickening character. The craters to these volcanoes are not found on the mountain top, but they are found in human mouths—in the antrum of Highmore, in the nasal passages, and externally on the face, neck, or even on the chest.

When the pulp of a tooth is dead and confined within its bony walls an outlet is sought, and must be affected for the escape of impure gases arising from the decomposing pulp and for the putrescent matter associated with it. When thus confined its only way of escape is through the dental foramen, and into tissues adjacent thereto. The pressure thus brought to bear upon the bony walls surrounding the apex of the root will in time perforate it at its weakest point, and the poisonous matter is forced through the opening thus formed and into the soft tissues, which soon yield to the pressure, and the imprisoned mass of corruption is liberated. The pain and swelling now subsides, but a dangerous nuisance has been created. The channel formed from the apex of the root to an external opening will not close while it is used for the passage of foul matter and gases that will flow unceasingly from the pulp canal.

The remedy of course is to rcmove the cause, and assist nature in affecting a cure, and to do this the pulp chamber must be opened, its contents removed, the canals cleansed and disinfected, the abscess healed, and the roots filled to the exclusion of all fluids and purulent matter. But how often this is not done. How many thousands of suffering mortals are to-day dragging out miserable lives because of these drainage tubes emptying themselves into the oval cavity—into the maxillary sinus or into the meatus of the nose. Such an abiding nuisance in the mouth cannot long exist without ruining health. But how few of the unfortunate sufferers realize the cause of their nervous irritability, their loss of appetite, their feeling of lassitude, their lack of energy, and their general prostration. And here let me say, that but few, in comparison to the number of these unfortunate sufferers seek relief at the hands of the dental practitioner. The patient is neither sick nor well, but debilitated and "good for nothing." The family physician is consulted, nervines and tonics are administered, but to no avail. The septic matter is vitiating the air that is breathed, and poisoning the food that is eaten.

The saliva that is poured into the mouth from the various glands must mingle with this poisonous matter and carry it into the stomach.

Sanitary means are being employed in all our cities at the present time, in view of the cholera scourge that it is feared will sweep over our land the coming summer. Our physicians wisely talk and write about the baneful influences of impure water, about miasma arising from the decomposition of vegetable matter, and about unwholesome food, and it would be well if the public would heed their timely warnings. And as dental practitioners, I feel that we, also have an important duty to perform, in enlightening our patients, and the public so far as we are able to do so, in the direction I have above indicated.

The subject is of paramount importance, and as the opportunities come to us in every day practice, let us not fail to impress upon the minds of our patient (when we find it necessary to do so), the fact that a clean mouth is essential to health.

The agitation of this subject, by the medical profession, is a step forward. Hitherto medical men have not given the matter the attention its importance demanded.

And now that this new light has dawned upon Dr. Sexton, it is not strange that, in hastily drawing his conclusions, he should have mingled much of error with the truths he has discovered. Possibly some of the cases that have come under his notice may have been the result of bad practice on the part of incompetent dental practitioners, but to charge the dental profession with their short-comings would be a matter of great injustice. Dr. Sexton is too hasty in his conclusions. First, he discovered that certain pulpless teeth had caused certain ailments, hence he condemns all pulpless teeth. He has discovered that certain dentists have failed to treat such teeth successfully, hence he condemns the dental profession for attempting to save teeth, it would be equally fair to condemn the whole medical profession, because of the incompetency of some of its members. But before dismissing the subject of pulpless teeth, it may be well for us to examine the subject a little more carefully from the standpoint of the medical writers above referred to. We cannot afford to make a mistake with regard to so important a matter. The higher a man stands in his profession, the more serious the mistakes he makes, and the more important it is that his practice be sound. An enthusiast or an extremist may injure a good cause. There are such men in our ranks.

A few years ago a prominent dentist said, "The tooth's pulp is its soul, and it is criminal to destroy it."

I heard another prominent dentist say, "If I find a part of the pulp dead, I amputate the dead tissues, and save the balance of the pulp alive."

A dentist has just moved away from Burlington, who has been in practice there for fifteen years, and during that time he has been using arsenic for obtunding sensitive dentine, and he has succeeded in accomplishing his purpose admirably. I have found in one month half a dozen filled teeth containing dead pulps, and, of course as many alveolar abscesses in active operation. The evils arising from such abominable methods of practice are simply appalling.

I have less frequently met with cases where those fistulous opening were on the neck or chest. In those cases the roots of the teeth are usually long, and when the abscess breaks through the lower border of the jaw, and the pus comes in contact with the soft tissues, it follows the course of the muscles and forms a sinous as it gravitates to some point on the neck or chest. I have known of a number of such cases being under medical treatment for years, where the affection was supposed to be of a strumpous nature, and the real cause was not suspected, and in every case a rapid recovery has followed the extraction of the offending tooth.

Gentlemen, I have no doubt but the most of you are disappointed in the nature of this paper. I have scarcely alluded to the treatment and filling of pulpless teeth. That had not been my purpose. But I have wished to call attention to the fact that a large majority of the ailments above referred to have be due to diseased teeth that have never received any attention whatever at the hands of competent dentists.

That pulpless teeth and roots may be treated, filled, and preserved in health in a majority of cases, is a settled question. Every well-informed dentist knows that to be a fact, the distinguished Dr. Sexton and the able editor of the *Medical Record* to the contrary notwithstanding.—*Iowa State Med. Reporter.*

